# *POPDC1* Variants Cause Atrioventricular Node Dysfunction and Arrhythmogenic Changes in Cardiac Electrophysiology and Intracellular Calcium Handling in Zebrafish

**DOI:** 10.3390/genes15030280

**Published:** 2024-02-23

**Authors:** Matthew R. Stoyek, Sarah E. Doane, Shannon E. Dallaire, Zachary D. Long, Jessica M. Ramia, Donovan L. Cassidy-Nolan, Kar-Lai Poon, Thomas Brand, T. Alexander Quinn

**Affiliations:** 1 Department of Physiology & Biophysics, Dalhousie University, Halifax, NS B3H 4R2, Canada; mstoyek@dal.ca (M.R.S.); sr571615@dal.ca (S.E.D.); shannondallaire@dal.ca (S.E.D.); zlong@dal.ca (Z.D.L.); jessicaramia@dal.ca (J.M.R.); dcn@dal.ca (D.L.C.-N.); 2 National Heart & Lung Institute, Imperial College London, London W12 0NN, UK; jialipan@gmail.com (K.-L.P.); t.brand@imperial.ac.uk (T.B.); 3 School of Biomedical Engineering, Dalhousie University, Halifax, NS B3H 4R2, Canada

**Keywords:** action potential, arrhythmias, autonomic nervous system, calcium transient, cyclic adenosine monophosphate (cAMP), heart rate, optogenetics, Popeye domain-containing (Popdc) genes

## Abstract

Popeye domain-containing (POPDC) proteins selectively bind cAMP and mediate cellular responses to sympathetic nervous system (SNS) stimulation. The first discovered human genetic variant (*POPDC1^S201F^*) is associated with atrioventricular (AV) block, which is exacerbated by increased SNS activity. Zebrafish carrying the homologous mutation (*popdc1^S191F^*) display a similar phenotype to humans. To investigate the impact of POPDC1 dysfunction on cardiac electrophysiology and intracellular calcium handling, homozygous *popdc1^S191F^* and *popdc1* knock-out (*popdc1^KO^*) zebrafish larvae and adult isolated *popdc1^S191F^* hearts were studied by functional fluorescent analysis. It was found that in *popdc1^S191F^* and *popdc1^KO^* larvae, heart rate (HR), AV delay, action potential (AP) and calcium transient (CaT) upstroke speed, and AP duration were less than in wild-type larvae, whereas CaT duration was greater. SNS stress by β-adrenergic receptor stimulation with isoproterenol increased HR, lengthened AV delay, slowed AP and CaT upstroke speed, and shortened AP and CaT duration, yet did not result in arrhythmias. In adult *popdc1^S191F^* zebrafish hearts, there was a higher incidence of AV block, slower AP upstroke speed, and longer AP duration compared to wild-type hearts, with no differences in CaT. SNS stress increased AV delay and led to further AV block in *popdc1^S191F^* hearts while decreasing AP and CaT duration. Overall, we have revealed that arrhythmogenic effects of POPDC1 dysfunction on cardiac electrophysiology and intracellular calcium handling in zebrafish are varied, but already present in early development, and that AV node dysfunction may underlie SNS-induced arrhythmogenesis associated with *popdc1* mutation in adults.

## 1. Introduction

The human heart beats around 100,000 times per day, with each beat initiated by an electrical impulse originating from the sinoatrial node (SAN). This impulse is conducted through the tissue of the atria (causing its excitation), passes through the atrioventricular node (AVN, where it is delayed), travels through the fast-conducting Purkinje system, and finally results in ventricular excitation. This well-coordinated sequence allows for efficient cardiac contraction, including time for blood from the atria to fill the ventricles before they are excited and contract (for a detailed description of the mechanisms involved in the proper spatio-temporal excitation and contraction of the heart see [1]). Disturbances of the heart’s electrical rhythmicity are known as arrhythmias, which can take the form of fast, slow, or irregular excitation. The causes of arrhythmias are broad-ranging, resulting from factors that include structural abnormalities (e.g., cardiomyopathy) and genetic mutations (e.g., channelopathies). Often, people unknowingly live with an underlying pro-arrhythmic condition (‘substrate’), and it is not until the occurrence of a specific stimulus (‘trigger’, such as increased sympathetic nervous system [SNS] activity) that an arrhythmia occurs (for an in-depth consideration of cardiac arrhythmogenesis, see [1,2]).

Autonomic inputs to the heart are critical for the regulation of its electrical and contractile activity, which is largely mediated by levels of cyclic adenosine monophosphate (cAMP) inside cardiomyocytes [3]. The Popeye domain-containing (POPDC) gene family encodes multi-compartment transmembrane proteins that selectively bind cAMP and mediate cellular responses to SNS stimulation [4,5]. Briefly, POPDC proteins are highly conserved and exist in both vertebrates and invertebrates (e.g., protostomes and cnidarians), indicating their fundamental physiological importance. They are composed of a short extracellular domain, three transmembrane domains, a carboxyterminal domain, and a highly conserved cytosolic Popeye domain, which resembles a high-affinity cAMP-binding domain. Three POPDC proteins are found in humans—POPDC1 (also known as blood vessel epicardial substance, BVES), POPDC2, and POPDC3, which are predominantly expressed in striated (cardiac and skeletal) muscle and are thought to exert their primary functional effects through interactions with proteins of the cAMP signaling pathway, along with ion channels and channel interacting proteins [6,7].

In cardiomyocytes, POPDC proteins are primarily localised to the sarcolemma and its specialised compartments (e.g., intercalated discs, transverse-tubules, caveolae, costameres) but have also been found at the nuclear envelope [4,5]. While all three POPDC genes are expressed in the mammalian heart, the highest expression levels are observed in the SAN and AVN, suggesting an important role in the initiation and conduction of electrical excitation [8]. Their interaction with structural, anchoring, and scaffolding proteins also makes POPDC an important modulator of the integrity of intercalated discs, which are essential for the mechanical and electrical coupling of adjacent cells [9,10]. As a result, cAMP-mediated influences of POPDC proteins on cardiac function are potentially multifaceted, and integral to the heart’s electrical activity through effects on action potential (AP) generation, electrical conduction, and intracellular calcium (Ca^2+^)-handling.

In humans, POPDC variants have been associated with cardiac electrical disturbances such as sick sinus syndrome [4], atrial fibrillation [11], AV block [12], and long QT syndrome [13]. The first variant of a POPDC gene discovered in humans (*POPDC1^S201F^*) was found in a family suffering from limb-girdle muscular dystrophy and SNS-induced AV block [12]. This recessive variant results in a substitution of the ultra-conserved serine 201 with a bulkier phenylalanine residue, reducing the binding affinity of POPDC1 for cAMP by 50%, while also impairing membrane trafficking of both POPDC1 and POPDC2. Several additional *POPDC1* variants have now been identified in patients [14,15,16,17,18,19,20], which similarly result in varying degrees of impaired POPDC membrane localisation, muscular dystrophy, and cardiac electrical dysfunction. While the specific mechanisms driving the cardiac effects of *POPDC1* variants are yet to be fully elucidated, dysfunction appears to relate to disturbances in the function, trafficking, localisation, and protein–protein interactions of POPDC1 with other membrane-associated proteins important for the heart’s electrical activity, as well as effects related to the cAMP signal transduction pathway [4].

In mice carrying knock-in or knock-out mutations of *Popdc1* subjected to physical or mental stress, as well as direct pharmacological β-adrenergic receptor (β-AR) stimulation (using the non-selective agonist isoproterenol, ISO), there is an age-dependent reduction in the normal SNS-induced increase in heart rate (HR) and temporary failures of SAN excitation (‘sinus pauses’ [8,12]). Introduction of a mutant variant homologous to human *POPDC1^S201F^* in zebrafish (*popdc1^S191F^*) results in a cardiac phenotype more closely resembling that observed in patients, including AV block, pericardial effusion, and impaired membrane trafficking of POPDC proteins [12]. The fact that the cardiac effects of POPDC1 dysfunction are more similar in zebrafish to humans than in mice may relate to greater similarities in cardiac function and relevant proteins between the two species, including comparable drivers of SAN automaticity, resting HR, sarcolemmal ion channels, AP morphologies, Ca^2+^-handling proteins, and intracardiac and extracardiac regulatory control [21,22,23,24,25]. In fact, the zebrafish is becoming an increasingly important experimental model for the study of cardiac (patho-)physiology [26,27,28]. The zebrafish has a fully sequenced genome, which may be altered using standard genetic techniques at a relatively low cost [29], and a majority of cardiac genes have human orthologs with analogous functions [30]. In addition, the zebrafish provides specific advantages for high throughput studies [31], as the whole heart can be visualised and interrogated in vivo in the transparent, externally developing embryo and larva [32,33] and ex vivo when isolated from the adult [21,22,34]. The similarity of the zebrafish *popdc1* mutant phenotype to humans, along with its advantages as an experimental model, suggests that zebrafish may be useful for studying the role of the *POPDC1* gene in the maintenance of normal cardiac rhythm and mechanisms by which POPDC1 dysfunction leads to SNS-induced cardiac arrhythmias.

The goal of the current study was to further investigate the effects of POPDC1 dysfunction on cardiac electrophysiology and intracellular Ca^2+^ handling using zebrafish. We assessed the effects of *popdc1^S191F^* or *popdc1* knock-out (*popdc1^KO^*) on the heart’s electrical function and Ca^2+^ handling in vivo in zebrafish larvae using fluorescent genetically expressed voltage- (GEVI) and Ca^2+^ (GECI) indicators and in ex vivo adult hearts using voltage- and Ca^2+^-sensitive fluorescent dyes. Overall, we show that the effects of POPDC1 dysfunction on cardiac electrophysiology and intracellular calcium handling are varied, but already present in early development, and that AV node dysfunction may underlie SNS-induced arrhythmogenesis in adult zebrafish hearts.

## 2. Materials and Methods

All experimental procedures were approved by the Dalhousie University Committee for Laboratory Animals and followed the guidelines of the Canadian Council on Animal Care.

### 2.1. Zebrafish Husbandry

**Figure 1:**
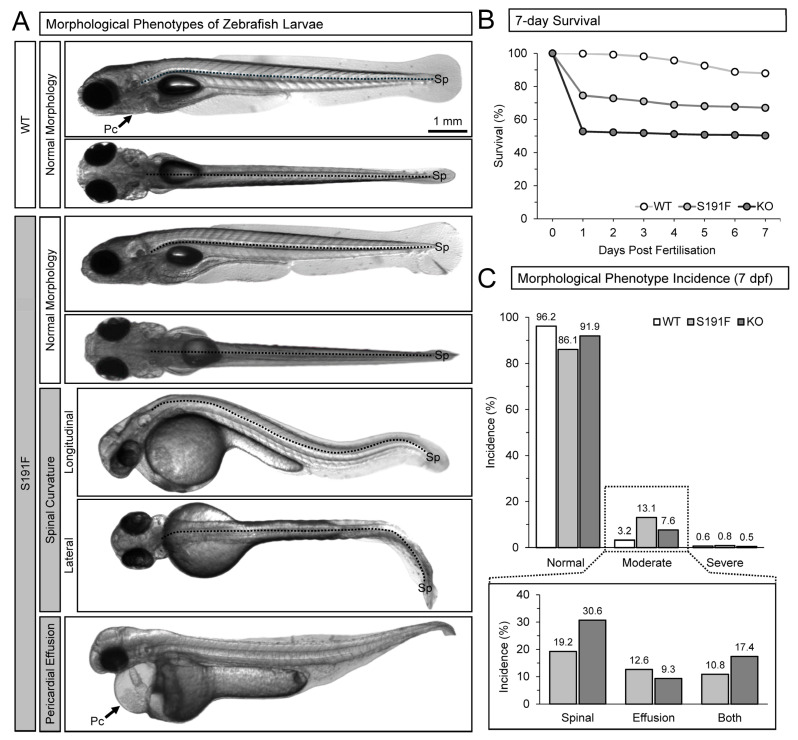
**Morphological phenotypes and survival of zebrafish larvae.** (**A**) Examples of 7 dpf WT and *popdc1^S191F^* (S191F) larvae with normal morphology (no phenotype) or a moderate morphological phenotype, involving spinal (Sp) curvature and/or pericardial (Pc) effusion. (**B**) Survival of WT (*n* = 2005 larvae, *N* = 22 clutches), *popdc1^S191F^* (*n* = 2469, *N* = 20), and *popdc1^KO^* (KO; *n* = 1740, *N* = 19) larvae over the first 7 dpf. (**C**) Incidence of larvae with normal morphology or a moderate or severe morphological phenotype (spinal curvature, pericardial effusion, or both) in surviving larvae (WT = 1774 survivors; *popdc1^S191F^* = 1670; *popdc1^KO^* = 879) at 7 dpf.

Zebrafish housing, breeding, and larval developmental staging were performed according to Westerfield [35]. Zebrafish were raised in the Faculty of Medicine Zebrafish CORE Facility at Dalhousie University. Larval experiments utilised healthy 7 days post fertilisation (dpf) wild-type (WT) larvae, and *popdc1^S191F^* and *popdc1^KO^* larvae with a normal morphology (no phenotype) or a moderate morphological phenotype (involving spinal curvature and/or pericardial effusion; examples shown in Figure 1A), maintained at 28 °C (physiological zebrafish temperature) in E3 solution (in mM: 5 NaCl, 0.17 KCl, 0.33 CaCl_2_, 0.33 MgSO_4_). To minimise possible confounding effects of overall health status, and to minimise potential pain and distress (according to ethical guidelines), any larvae displaying a severe morphological phenotype (i.e., spinal curvature that affected normal motility and/or pericardial effusion that affected normal cardiac chamber development) were removed, euthanised, and considered to not have survived. Survival from 1 to 7 dpf and the incidence of morphological phenotypes at 7 dpf were measured for WT, *popdc1^S191F^*, and *popdc1^KO^* larvae (discussed in Results and present in Figure 1B,C).

Adult experiments utilised hearts isolated from a mixed-sex population of 18–22 months post fertilisation (mpf) zebrafish maintained in a commercial recirculation system (Aquatic Habitats, Pentair, MN, USA) in conditioned water.

### 2.2. Zebrafish Strains

Larval experiments utilised zebrafish expressing the FRET-based GEVI Mermaid (green-emitting fluorescent donor [mUKG] and orange-emitting fluorescent acceptor [mKOκ]) [36] or the GFP-calmodulin-based GECI GCaMP3 [37], driven by the myosin light chain kinase regulatory region (*myl7*) promoter for cardiac-specific expression, including: WT [*Tg(myl7:Mermaid)*, *Tg(myl7:GCaMP3)*] and homozygote *popdc1^S191F^* [*Tg(popdc1^S191F^; myl7:Mermaid)*, *Tg(popdc1^S191F^; myl7:GCaMP3)*] on a *casper* background and *popdc1^KO^* [*Tg(popdc1^KO^; myl7:Mermaid)*, *Tg(popdc1^KO^; myl7:GCaMP3*)] on AB background. Adult isolated heart experiments utilised WT or homozygote *popdc1^S191F^* AB zebrafish.

### 2.3. Generation of popdc1^KO^ Zebrafish

Generation of the *popdc1^S191F^* mutant has been previously reported [12]. A *popdc1^KO^* mutant zebrafish line was generated using the GoldyTALEN-modified scaffold [38]. The left TALEN (P1E2_29TALL: 5′-CCAGTGTTCCAGCT-3′) and right TALEN (P1E2_29TALR: 5′-CCTGGCATAGTGTGGCG-3′) were assembled via the GoldenGate method. For ease of analysis, TALEN recognition sequences flanked a *Bam*HI restriction site in exon 2 of *popdc1*, which was destroyed upon successful targeting. TALEN repeat variable di-residues (RVDs) were cloned into a pT3TS-driven TALEN scaffold. The resulting mRNA was injected into 1-cell-stage zebrafish embryos. Larvae were molecularly tested at 2 dpf or raised to adulthood for germline mutation analysis. Somatic and germline TALEN-induced mutations were evaluated by PCR and restriction fragment length polymorphisms. The primer pair P1TALE2_491F: 5′-GTTATATTTTCACGCCACTACGTT-3′ and P1TALE2_988R: 5′-TACCCTATAAACACAAGCGGAT-3′ was used to amplify the locus. Subsequently, the PCR fragment was restriction-digested with *BamH*I. The mutant allele produced a single 519-bp fragment, while the WT allele produced 2 fragments of 314 and 205 bp. The mutant line was backcrossed six times with the AB line in order to outcross any off-target mutations. Homozygous mutant and WT animals were bred separately. The mutant allele carries a 10 bp deletion (nt 45–55) in exon 2 of *popdc1*. This results in a frameshift and a premature stop codon. A 36 amino acid long peptide is predicted to be encoded by the mutant transcript (amino acid sequence: MSNTTSALPSSVPAVQTPHYARIGNSLITCCSTWPT*; the underlined sequence is unique to the mutant allele).

### 2.4. Larval Zebrafish Preparation and Functional Imaging

Prior to imaging, larvae were incubated for 90 min in E3 solution with 100 µM (S)-3′-amino blebbistatin (24170, Cayman Chemical, Ann Arbor, MI, USA), which is a more soluble, more stable, and less phototoxic (during blue wavelength light exposure needed for fluorescent imaging) form of the excitation uncoupler blebbistatin [39], to minimise imaging artefacts associated with cardiac contraction. Zebrafish were then transferred to an imaging dish lined with black Sylgard (DC 170, Dow Corning, Midland, TX, USA) under a layer of 3% methylcellulose (M0387, Sigma-Aldrich, St. Louis, MO, USA) and positioned in a left-lateral-side-up orientation for an optimal view of both heart chambers. The observation dish was filled with an E3 solution containing 0.35 mM tricaine (MS-222; E10521, Sigma-Aldrich, which provides adequate anesthesia, while causing no effects on HR or cardiac mechanical function [40]) and temperature-controlled at 28 °C (TC-344C, Warner Instruments) with a warmed platform (WP-16, Warner Instruments, Hamden, CT, USA) for the duration of the experiment. Larvae were left to acclimatise for 5 min, followed by a 10 s recording of membrane voltage (V_m_) or intracellular Ca^2+^ dynamics.

**Figure 2:**
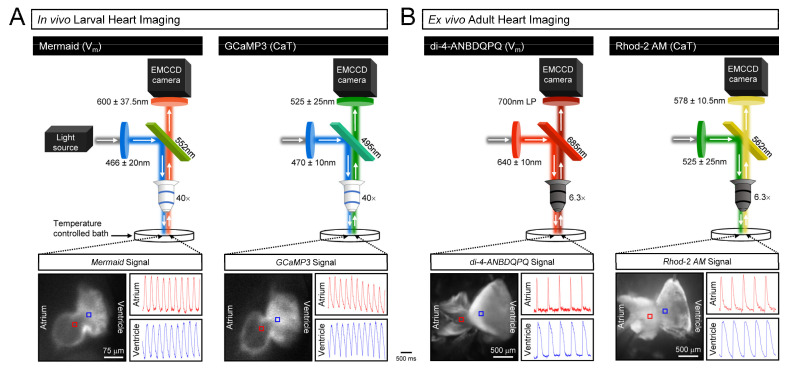
**Imaging setup.** Schematics of the (**A**) in vivo larval and (**B**) ex vivo adult heart imaging setups, along with images of hearts from 7 dpf and adult WT zebrafish and representative fluorescent V_m_ and Ca^2+^ signals recorded from the atrium and ventricle.

In vivo functional imaging of V_m_ or Ca^2+^ in zebrafish larvae was performed as previously described [41]. Larvae were epi-illuminated with a mercury arc lamp (U-HGLPS, Olympus, Tokyo, Japan) through a compound upright fluorescent microscope (BX63, Olympus) with a 0.80 NA, 26.5 FN, 40×, infinity-corrected water immersion objective (LUMPlanFLN, Olympus). The mUKG donor of the GEVI (Mermaid) was excited with light passed through a 466 ± 20 nm filter (FF01-466/40, Semrock, Lake Forest, CA, USA) and fluorescence emitted from the mKOκ acceptor reflected with a 552 nm dichroic filter (FF552-Di02, Semrock) and passed through a 600 ± 37.5 nm filter (HQ600/75M, Chroma Technology, Bellows Falls). The GECI (GCaMP3) was excited with light passed through a 470 ± 10 nm filter (D470/20X, Chroma Technology) and emitted fluorescence was reflected with 495 nm dichroic filter (FF495-Di03, Semrock) and passed through a 525 ± 25 nm filter (FF03-525/50, Semrock). Each period of light exposure was 10 s, with fluorescence recorded with a 128 × 128 pixel, 16-bit electron-multiplying charge-coupled device (EMCCD) camera (iXon3, Andor Technology, Belfast, UK) at 500 frames/s using Solis software (Andor Technology). A schematic of the imaging setup, along with representative images of 7 dpf larvae hearts and V_m_ and Ca^2+^ signals recorded from the atrium and ventricle, can be seen in Figure 2A.

### 2.5. Ex Vivo Adult Heart Preparation and Functional Imaging

For adult heart experiments, hearts were removed from the animal and instrumented as previously described [22]. Zebrafish were euthanised with 1.5 mM tricaine in Tris-buffered (pH 7.4; BP152, Thermo Fisher Scientific, Waltham, MA, USA) room temperature tank water until they showed no opercular respiration movements and lacked a locomotor response to fin pinch (~2 min). They were then placed in a Sylgard-lined Petri dish filled with HEPES-buffered saline solution (in mM: 135 NaCl, 5 KCl, 5.5 NaHCO_3_; 1.5 NaH_2_PO_4_; 1.7 MgCl_2_; 1.8 CaCl_2_; 7.5 Glucose, 5 Creatine, 10 HEPES [42]), with an osmolality of 300 ± 5 mOsm/kg and a pH of ~7.30 ± 0.05 at 28 °C. A ventral midline incision was made through the body wall and pericardium to expose the heart, which was removed via incisions at the sinus venosus (venous pole) and the ventral aorta (arterial pole). The isolated heart was placed in a Sylgard-lined Petri dish with 5 mL of the saline solution and maintained at 28.0 ± 0.5 °C, with temperature monitored by a thermocouple (T-type Pod, ADInstruments, Sydney, Australia). The heart was secured in the dish with 100 µm pins (Fine Science Tools, Foster City, CA, USA) at the arterial and venous poles of the heart. Custom suction electrodes were placed on the atrium and ventricle and connected to ECG amplifiers (Animal Bio Amp, ADInstruments). Temperature and ECG signals were continuously recorded for the entire experiment at 2 kHz using a data acquisition device (PowerLab, ADInstruments) controlled by LabChart (ADInstruments). Hearts that could not maintain 1:1 AV conduction when paced at 2 Hz were excluded from further experimentation and analysis.

Prior to V_m_ imaging, hearts were incubated in saline solution with 10 µM of the excitation-contraction uncoupler (±)-blebbistatin (BB592490, Toronto Research Chemicals, Toronto, Canada) to minimise imaging artefacts associated with cardiac contraction. After 30 min, 10 µM of the V_m_-sensitive dye di-4-ANBDQPQ (Potentiometric Probes) was added to the bath as a concentrated (35.1 mM) bolus over the heart, gently mixed, and left for 15 min to allow for dye loading and cessation of contraction. The solution was then replaced with saline solution containing 10 µM ± blebbistatin. Prior to Ca^2+^ imaging, hearts were incubated in saline solution with 0.02% of the surfactant Pluronic F-127 (P2443, Sigma-Aldrich) to facilitate dye loading. After 1 min, 10 µM of the Ca^2+^-sensitive fluorescent dye Rhod-2 AM (ab1427870, Abcam) was added to the bath as a concentrated (1 mM) bolus over the heart, gently mixed, and after an additional 1 min, 10 µM ±blebbistatin was added. After 15 min, the solution was replaced with saline solution containing 10 µM ±blebbistatin and 1 mM of the organic anion transporter inhibitor probenecid (P36400, Fisher Scientific) to minimise dye extrusion, and left for 30 min to allow for Rhod-2 de-esterification and cessation of contraction. Hearts were then paced at 2 Hz for the remainder of the experiment to control for HR, using a square pulse stimulator (S48, Grass Instruments, Quincy, MA, USA) with a 2 ms pulse duration and current ranging from 20–100 μA.

Ex vivo functional imaging of V_m_ and Ca^2+^ in isolated hearts was performed as previously described [34]. Hearts were epi-illuminated with the mercury arc lamp through a compound macroscope (MVX10, Olympus) with a 1× objective (MV PLAPO 1×, Olympus) at 6.3× magnification. The V_m_-sensitive dye was excited with light passed through a 640 ± 10 nm filter (D640/20X, Chroma Technology) and emitted fluorescence was reflected with a 685 nm dichroic filter (FF685-Di02, Semrock) and passed through a 700 nm long-pass filter (700LP, Chroma Technology). The Ca^2+^ dye was excited with light passed through a 525 ± 25 nm filter (FF03-525/50, Semrock) and emitted fluorescence was reflected with a 562 nm dichroic filter (FF562-Di03, Semrock) and passed through a 578 ± 10.5 nm filter (FF01-578/21, Semrock). Each period of light exposure was 5 s, with fluorescence recorded with the EMCCD camera and Solis software at 500 frames/s. A schematic of the imaging setup, along with representative images of ex vivo adult hearts and V_m_ and Ca^2+^ signals recorded from the atrium and ventricle, can be seen in Figure 2B.

Importantly, unlike mammalian hearts, in which fluorescent signals are collected primarily from the superficial epicardium due to the limited penetration of light into tissue, the adult zebrafish heart has relatively thin atrial and ventricular walls (~100–200 μm [43]). Myocardial penetration of the green light used to excite the Ca^2+^-sensitive dye in our experiments (525 ± 25 nm) has been shown by others to be ~50% at 1 mm [44,45] with the red light used to excite the V_m_-sensitive dye penetrating even further, so both signals will come from a bulk of the atrial and ventricular wall.

### 2.6. SNS Stress

Zebrafish larvae and adult hearts were exposed to 100 µM ISO (I6504, Sigma-Aldrich) to simulate increased SNS activity. For larvae, the concentration was based on a previous report, which demonstrated arrhythmogenic effects of 100 µM ISO in *popdc1^S191F^* larval zebrafish [12]. For adult hearts, previous reports have demonstrated that 40 µM ISO results in a physiological (~33%) increase in HR [22]. As our aim in the current study was to induce SNS stress, we used a 2.5× higher concentration (100 µM), which also matched with the larval experiments. Larvae were incubated with ISO for 5 min before V_m_ or Ca^2+^ imaging. In adult hearts, fluorescence was recorded immediately before and 0.5, 1.0, 3.0, and 5.0 min after ISO was added to the bath.

### 2.7. AP and Ca^2+^ Transient Measurements

Fluorescent recordings were analysed using custom routines in Matlab (MathWorks, Natick, MA, USA). For recordings in larvae, a 50 Hz low-pass Butterworth filter and a 5 × 5 pixel moving box filter were applied. A 5 × 5 pixel region of interest (ROI) was chosen on the atrium and ventricle near the AVN. Ten consecutive AP or Ca^2+^ transients (CaT) were selected and beat-to-beat measurements of HR (calculated from the peak-to-peak time), AV delay (calculated as the time of activation between the atrium and ventricle), AP or CaT upstroke speed (dF_n_/dt_max_, calculated as the maximum change in normalised fluorescence over change in time), and AP duration or CaT duration at 80% recovery (APD_80_ or CaTD_80_, calculated as the time from the point of dF_n_/dt_max_ to an 80% decline in fluorescence from peak to rest) were averaged over the beats. For recordings in adult hearts, a 50 Hz low-pass Butterworth filter was applied. A 10 × 10 pixel ROI was chosen at the mid-atrium and ventricle near the AVN. Ten consecutive AP or CaT were selected and signal-averaged by aligning the points of dF_n_/dt_max_. Measurements of AV delay, AP, or CaT dF_n_/dt_max_, and APD_80_ or CaTD_80_ were then calculated from the averaged AP or CaT.

### 2.8. Statistical Analysis

Values are reported as mean ± standard error of the mean (SEM). Statistical analysis was performed in Prism (GraphPad, New York, NY, USA). Groups were compared by paired or unpaired two-tailed Student’s *t*-test or the non-parametric Kruskal–Wallis test (as results were generally not normally distributed) with Dunn’s corrected post hoc tests for multiple comparisons, as appropriate. Significance was indicated by *p* < 0.05.

## 3. Results

### 3.1. Effects of popdc1 Dysfunction on Larval Survival and Incidence of Morphological Phenotypes

Effects of *popdc1* dysfunction on survival from 1 to 7 dpf and the incidence of morphological phenotypes at 7 dpf were measured for WT (*n* = 2005 larvae; *N* = 22 clutches), *popdc1^S191F^* (*n* = 2469, *N* = 20), and *popdc1^KO^* (*n* = 1740, *N* = 19) larvae. Results are presented in Figure 1B,C.

Between 0 and 1 dpf, there was considerable die-off or development of a severe morphological phenotype (requiring euthanasia) of *popdc1^S191F^* and *popdc1^KO^* larvae, such that at 1 dpf, they each had lower survival than WT (74.5% and 52.8% vs. 99.9%), which persisted until 7 dpf (67.1% and 50.3% vs. 87.9%). In the *popdc1^S191F^* and *popdc1^KO^* larvae that survived to 7 dpf, there was a 13.1% (218 of 1670 survivors) and 7.6% (67 of 879) incidence of a moderate morphological phenotype, which was 4.1× and 2.4× greater than the incidence in WT (3.2%; 57 of 1774).

### 3.2. Effects of popdc1 Dysfunction on HR and AVN Function in Larval Zebrafish

**Figure 3:**
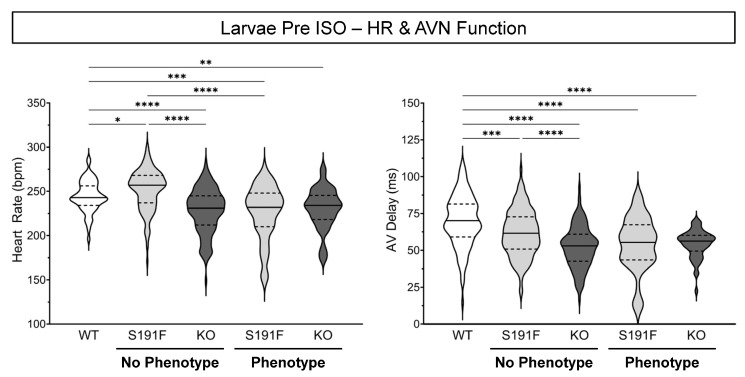
**Effects of *popdc1* dysfunction on HR and AVN function in larval zebrafish.** HR (**left**) and AV delay (**right**) in WT (*n* = 130, *N* = 6), *popdc1^S191F^* (S191F), and *popdc1^KO^* (KO) zebrafish larvae with no morphological phenotype (*n* = 137 and *n* = 143, *N* = 6) or a moderate phenotype (*n* = 69 and *n* = 57, *N* = 6). Groups were compared by the non-parametric Kruskal–Wallis test, with Dunn’s corrected post hoc tests for multiple comparisons. * *p* < 0.05, ** *p* < 0.01, *** *p* < 0.001, **** *p* < 0.0001.

Effects of *popdc1* dysfunction on basal HR and AV delay were investigated in 7 dpf WT larvae (*n* = 130, *N* = 6) and *popdc1^S191F^* and *popdc1^KO^* larvae with no morphological phenotype (*n* = 137 and *n* = 143, *N* = 6) or a moderate phenotype (*n* = 69 and *n* = 57, *N* = 6). Results are presented in Figure 3 and Table 1.

In larvae with no morphological phenotype, HR was higher in *popdc1^S191F^* than WT *(p* = 0.0396), while it was lower in *popdc1^KO^* than both WT and *popdc1^S191F^* (*p* < 0.0001 for both). In larvae with a phenotype, HR was lower in both *popdc1^S191F^* and *popdc1^KO^* than WT (*p* = 0.0007, *p* = 0.0034), and lower in *popdc1^S191F^* with a phenotype than with no phenotype (*p* < 0.0001). In *popdc1^S191F^* and *popdc1^KO^* larvae with no morphological phenotype (*p* = 0.0010, *p* < 0.0001) or a phenotype (*p* < 0.0001 for both), AV delay was greater than WT. In larvae with no phenotype, AV delay in *popdc1^S191F^* was greater than *popdc1^KO^* (*p* < 0.0001).

### 3.3. Effects of popdc1 Dysfunction on V_m_ Dynamics in Larval Zebrafish

**Figure 4:**
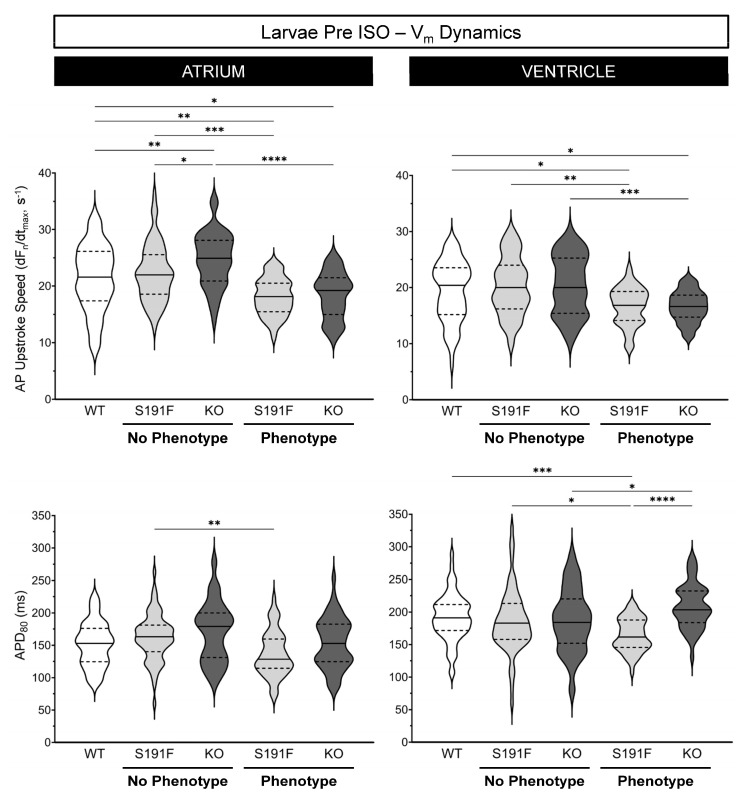
**Effects of *popdc1* dysfunction on V_m_ dynamics in larval zebrafish.** Atrial (**left column**) and ventricular (**right column**) AP upstroke speed (**upper row**) and APD_80_ (**lower row**) in WT (*n* = 69, *N* = 4), *popdc1^S191F^* (S191F), and *popdc1^KO^* (KO) zebrafish larvae with no morphological phenotype (*n* = 69 and *n* = 59, *N* = 4) or a moderate phenotype (*n* = 50 and *n* = 50, *N* = 5). Groups were compared by the non-parametric Kruskal–Wallis test, with Dunn’s corrected post hoc tests for multiple comparisons. * *p* < 0.05, ** *p* < 0.01, *** *p* < 0.001, **** *p* < 0.0001.

Effects of *popdc1* dysfunction on atrial and ventricular AP upstroke speed and APD_80_ were investigated in WT larvae (*n* = 69, *N* = 4) and *popdc1^S191F^* and *popdc1^KO^* larvae with no morphological phenotype (*n* = 69 and *n* = 59, *N* = 4) or a moderate phenotype (*n* = 50 and *n* = 50, *N* = 5). Results are presented in Figure 4 and Table 1.

In larvae with no morphological phenotype, atrial AP upstroke speed was faster in *popdc1^KO^* than both WT and *popdc1^S191F^* (*p* = 0.0049, *p* = 0.0483). In larvae with a phenotype, it was slower in both *popdc1^S191F^* and *popdc1^KO^* than WT (*p* = 0.0016, *p* = 0.0106) and larvae with no phenotype (*p* = 0.0001, *p* < 0.0001). In the ventricle, *popdc1^S191F^* and *popdc1^KO^* larvae with a phenotype had a slower AP upstroke speed than WT (*p* = 0.0251, *p* = 0.0110) or larvae with no phenotype (*p* = 0.0019, *p* = 0.0007).

In the atrium, APD_80_ was shorter in *popdc1^S191F^* larvae with a phenotype than with no phenotype (*p* = 0.0023). In the ventricle, APD_80_ was shorter in *popdc1^S191F^* with a phenotype than WT, *popdc1^S191F^* with no phenotype, and *popdc1^KO^* with a phenotype (*p* = 0.0004, *p* = 0.0284, *p* < 0.0001), which was longer than *popdc1^KO^* without a phenotype (*p* = 0.0321).

Comparing the cardiac chambers, AP upstroke speed was slower and APD_80_ was longer in the ventricle than the atrium in all groups (*p* < 0.0001 for all).

### 3.4. Effects of popdc1 Dysfunction on Ca^2+^ Dynamics in Larval Zebrafish

**Figure 5:**
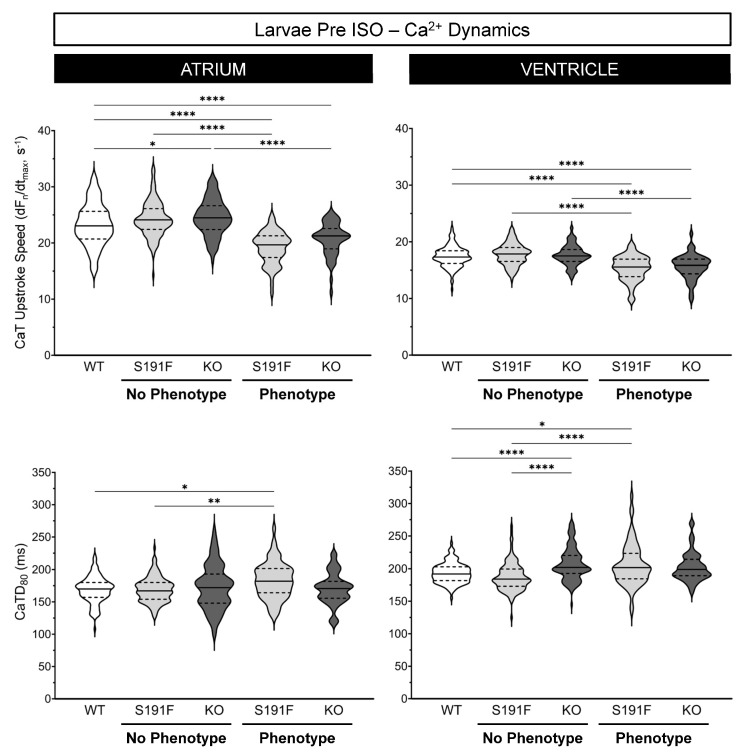
**Effects of *popdc1* dysfunction on Ca^2+^ dynamics in larval zebrafish.** Atrial (**left column**) and ventricular (**right column**) CaT upstroke speed (**upper row**) and CaTD_80_ (**lower row**) in WT (*n* = 130, *N* = 6), *popdc1^S191F^* (S191F), and *popdc1^KO^* (KO) zebrafish larvae with no morphological phenotype (*n* = 137 and *n* = 143, *N* = 6) or a moderate phenotype (*n* = 67 and *n* = 57, *N* = 5). Groups were compared by the non-parametric Kruskal–Wallis test, with Dunn’s corrected post hoc tests for multiple comparisons. * *p* < 0.05, ** *p* < 0.01, **** *p* < 0.0001.

Effects of *popdc1* dysfunction on atrial and ventricular CaT upstroke speed and CaTD_80_ were investigated in WT larvae (*n* = 130, *N* = 6) and *popdc1^S191F^* and *popdc1^KO^* larvae with no morphological phenotype (*n* = 137 and *n* = 143, *N* = 6) or a moderate phenotype (*n* = 67 and *n* = 57, *N* = 5). Results are presented in Figure 5 and Table 1.

### 3.5. Cardiac Effects of SNS Stress in Larval Zebrafish with popdc1 Dysfunction

**Figure 6:**
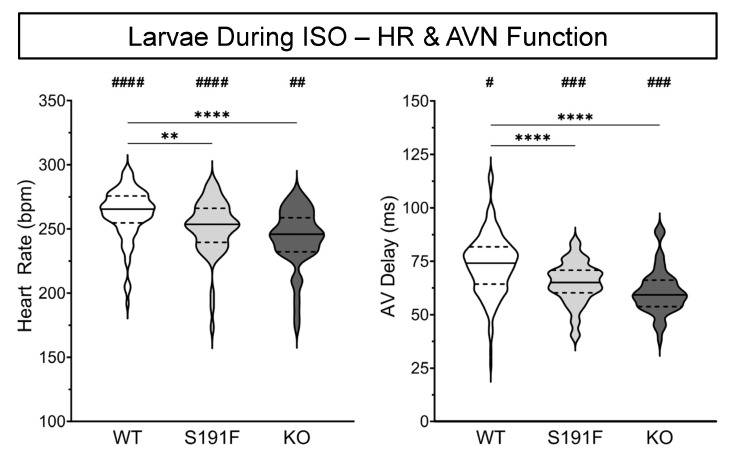
**Effects of ISO on HR and AVN function in larval zebrafish.** HR (**left**) and AV delay (**right**) in WT (*n* = 96, *N* = 5), *popdc1^S191F^* (S191F; *n* = 53, *N* = 5), and *popdc1^KO^* (KO; *n* = 95, *N* = 5) zebrafish larvae with a moderate morphological phenotype. Groups were compared by the non-parametric Kruskal–Wallis test, with Dunn’s corrected post hoc tests for multiple comparisons (** *p* < 0.01, **** *p* < 0.0001), and to values pre-ISO (from Figure 3) using unpaired two-tailed Student’s *t*-tests (^#^ *p* < 0.05, ^##^ *p* < 0.01, ^###^ *p* < 0.001, ^####^ *p* < 0.0001).

**Figure 7:**
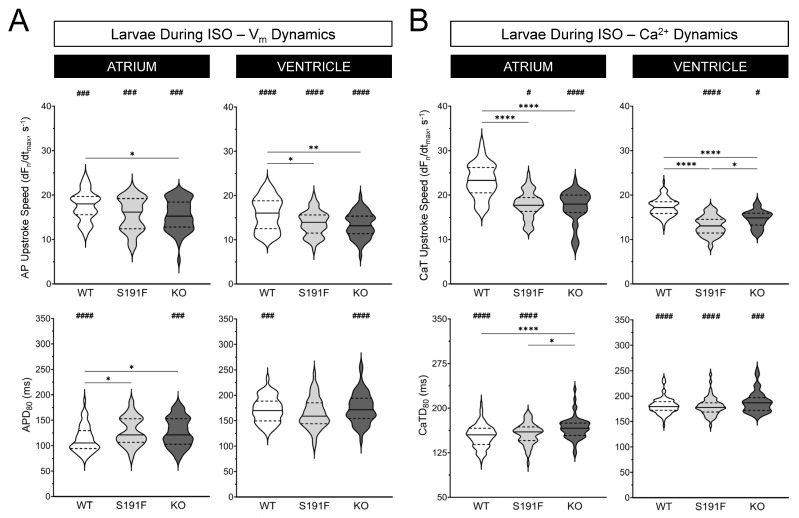
**Effects of ISO on V_m_ and Ca^2+^ dynamics in larval zebrafish.** (**A**) AP upstroke speed (**upper row**) and APD_80_ (**lower row**) and (**B**) CaT upstroke speed (**upper row**) and CaTD_80_ (**lower row**) in WT (*n* = 58 and *n* = 54, *N* = 5), *popdc1^S191F^* (S191F; *n* = 47 and *n* = 56, *N* = 5), and *popdc1^KO^* (KO; *n* = 55 and *n* = 54, *N* = 5) from the atrium (left column) and ventricle (right column) of zebrafish larvae with a moderate morphological phenotype. Groups were compared by the non-parametric Kruskal–Wallis test, with Dunn’s corrected post hoc tests for multiple comparisons (* *p* < 0.05, ** *p* < 0.01, **** *p* < 0.0001), and to values pre-ISO (from Figure 4 and Figure 5) using unpaired two-tailed Student’s *t*-tests (^#^ *p* < 0.05, ^###^ *p* < 0.001, ^####^ *p* < 0.0001).

To investigate the cardiac effects of SNS stress in zebrafish with *popdc1* dysfunction, WT larvae and *popdc1^S191F^* and *popdc1^KO^* larvae with a morphological phenotype were exposed to 100 μM ISO (as previously reported [12]), and effects on HR and AV delay (*n* = 96, *n* = 53, and *n* = 95, *N* = 5), atrial and ventricular AP upstroke speed and APD_80_ (*n* = 58, *n* = 47, and *n* = 55, *N* = 5), and CaT upstroke speed and CaTD_80_ (*n* = 54, *n* = 56, and *n* = 54, *N* = 5) were assessed. Results are presented in Figure 6 and Figure 7 and Table 2.

Figure 6 shows that HR and AV delay were lower in *popdc1^S191F^* (*p* < 0.0001 for both) and *popdc1^KO^* (*p* = 0.0396, *p* < 0.0001) than WT, and that both were increased by ISO in all three groups (*p* < 0.0001, *p* = 0.0065, *p* < 0.0001 and *p* = 0.0003, *p* = 0.0009, *p* = 0.0451; compared to data found in Figure 3).

Figure 7 shows that AP upstroke speed in the atrium was slower in *popdc1^KO^* than in WT (*p* < 0.0173), and that it was increased by ISO in all three groups (*p* = 0.0001, *p* = 0.0006, *p* = 0.0001). APD_80_ in both *popdc1^S191F^* and *popdc1^KO^* (*p* = 0.0138, *p* = 0.0190) was shorter than WT and was decreased by ISO in WT and *popdc1^KO^* (*p* < 0.0001, *p* = 0.0006). In the ventricle, AP upstroke speed in *popdc1^S191F^* and *popdc1^KO^* (*p* = 0.0147, *p* = 0.0012) was slower than in WT, and was decreased by ISO in all three groups (*p* < 0.0001 for each). There were no baseline differences in APD_80_, although it was decreased by ISO in WT and *popdc1^KO^* (*p* = 0.0004, *p* < 0.0001).

CaT upstroke speed in the atrium was slower in *popdc1^S191F^* and *popdc1^KO^* than WT (*p* < 0.0001 for both) and was decreased by ISO in *popdc1^S191F^* and *popdc1^KO^* (*p* = 0.0266, *p* < 0.0001). CaTD_80_ in *popdc1^KO^* was longer than WT (*p* < 0.0001), which itself was longer than *popdc1^S191F^* (*p* = 0.0355), and it was decreased by ISO in WT and *popdc1^S191F^* (*p* < 0.0001 for both). In the ventricle, CaT upstroke speed in *popdc1^S191F^* and *popdc1^KO^* was slower than WT (*p* < 0.0001 for both), was slower in *popdc1^S191F^* than *popdc1^KO^* (*p* = 0.0151), and was decreased by ISO in *popdc1^S191F^* and *popdc1^KO^* (*p* < 0.0001, *p* = 0.0101). There were no baseline differences in CaTD_80_, although it was decreased by ISO in all three groups (*p* < 0.0001, *p* < 0.0001, *p* = 0.0008).

### 3.6. Cardiac Effects of popdc1 Dysfunction and SNS Stress in Adult Isolated Hearts

**Figure 8:**
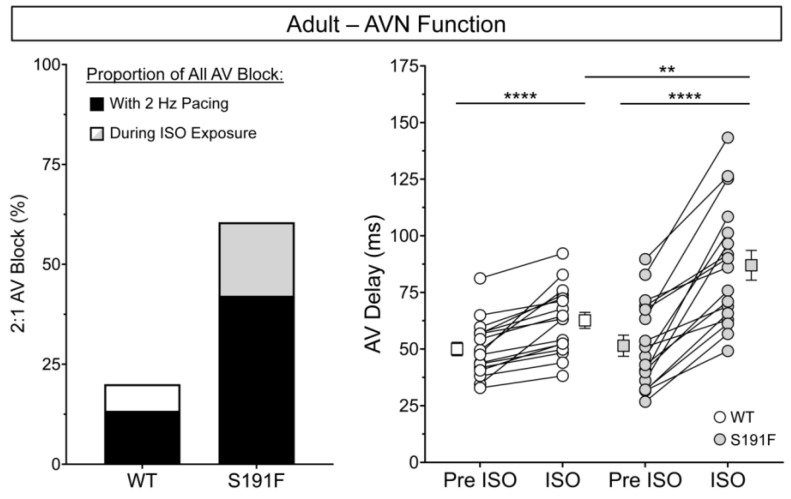
**Effects of ISO on AVN function in adult isolated zebrafish hearts.** Incidence of 2:1 AV block in ex vivo hearts isolated from WT (*n* = 18) and *popdc1^S191F^* (S191F; *n* = 18) adult zebrafish (**left**) and changes in AV delay during ISO exposure (**right**). Groups were compared by paired and unpaired two-tailed Student’s *t*-tests (** *p* < 0.01, **** *p* < 0.0001).

**Figure 9:**
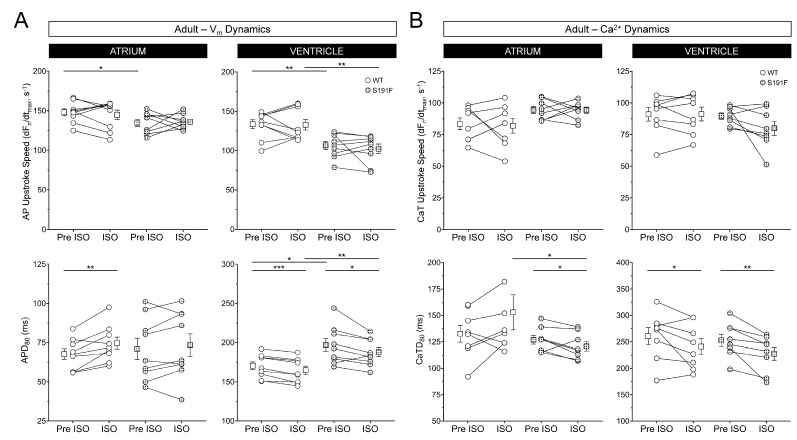
**Effects of ISO on V_m_ and Ca^2+^ dynamics in adult isolated zebrafish hearts.** (**A**) AP upstroke speed (**upper row**) and APD_80_ (**lower row**) and (**B**) CaT upstroke speed (**upper row**) and CaTD_80_ (**lower row**) in ex vivo hearts isolated from WT (*n* = 9) and *popdc1^S191F^* (S191F; *n* = 9) adult zebrafish before (Pre) and during ISO exposure from the atrium (left column) and ventricle (right column). Groups were compared by paired and unpaired two-tailed Student’s *t*-tests (* *p* < 0.05, ** *p* < 0.01, *** *p* < 0.001).

The effects of SNS stress on AV function and V_m_ and Ca^2+^ dynamics were further investigated by exposing ex vivo hearts isolated from WT (*n* = 9) and *popdc1^S191F^* (*n* = 9) adult zebrafish to ISO. Results are presented in Figure 8 and Figure 9 and Table 3.

Figure 8 shows that, when comparing all isolated hearts (including those that developed 2:1 AV block with the initiation of 2 Hz pacing, so were excluded from further study), there was a higher incidence of 2:1 AV block in *popdc1^S191F^* than WT. In total, 23 of 38 *popdc1^S191F^* hearts (60.5%) developed AV block (either with the initiation of 2 Hz pacing or after 5 min of ISO exposure), compared to 6 of 30 WT hearts (20.0%). For *popdc1^S191F^* hearts, this included 16 of 38 (42.1%) with 2 Hz pacing and 7 of 18 (38.9%) during ISO, compared to 4 of 30 (13.3%) and 2 of 18 (11.1%) WT hearts.

The difference in the incidence of AV block during ISO exposure between *popdc1^S191F^* and WT was associated with a difference in the effect of ISO on AV delay. AV delay was increased in both groups (*p* < 0.0001 for both), but to a greater extent in *popdc1^S191F^*, so that it became greater in *popdc1^S191F^* than WT (*p* = 0.0026).

Figure 9 shows that the effects of ISO on V_m_ and Ca^2+^ dynamics were varied in adult hearts. In both the atrium and ventricle, AP upstroke speed was slower at baseline in *popdc1^S191F^* than WT (*p* = 0.0478, *p* = 0.0029), and remained so in the ventricle during ISO exposure (*p* = 0.0037). In the ventricle of *popdc1^S191F^*, APD_80_ was longer than WT, both before and during ISO (*p* = 0.0132, *p* = 0.0084). ISO had differential effects on APD_80_ in the atrium and ventricle, as APD_80_ was increased by ISO in the atrium of WT (*p* = 0.0062), while it was decreased in the ventricle of both WT and *popdc1^S191F^* (*p* = 0.0004, *p* = 0.0297). In contrast, there were no significant differences at baseline in CaT upstroke speed or CaTD_80_ in the atrium or ventricle of WT compared to *popdc1^S191F^*. ISO had no significant effect on atrial or ventricular CaT upstroke speed but had differential effects on CaTD_80_ in WT compared to *popdc1^S191F^*. In the atrium, CaTD_80_ was decreased by ISO in *popdc1^S191F^* only (*p* = 0.0284), such that during ISO, CaTD_80_ was shorter in *popdc1^S191F^* than WT (*p* = 0.0416). In the ventricle, CaTD_80_ was decreased by ISO in both WT and *popdc1^S191F^* (*p* = 0.0415, *p* = 0.0039).

Comparing the cardiac chambers, AP upstroke speed was slower in the ventricle than the atrium in both WT and *popdc1^S191F^* (*p* = 0.0482, *p* = 0.0006), while APD_80_ and CaTD_80_ were longer in the ventricle than the atrium in both WT and *popdc1^S191F^* (*p <* 0.0001 for all).

## 4. Discussion

Much is now known about the expression, function, and physiological importance of POPDC proteins, particularly in relation to the pathophysiological consequences of its dysfunction in the heart [4]. The first human variant of *POPDC1* discovered in patients (*POPDC1^S201F^*) results in impaired membrane trafficking of both POPDC1 and POPDC2, a reduction in the protein’s affinity for cAMP, and SNS-induced 2:1 AV block [12]. In mice, mutation or knock-out of *Popdc1* results in age-dependent sinus pauses during physical-, mental-, or direct SNS-induced stress [8,12], while zebrafish carrying the *popdc1^S191F^* mutation (which is homologous to *POPDC1^S201F^*), like human, exhibit susceptibility to 2:1 AV block [12]. This similarity in phenotype suggests that the zebrafish may be a useful experimental model to study mechanisms of POPDC1-induced cardiac dysfunction.

Here, we sought to further establish the zebrafish for this purpose, by defining the effects of *popdc1^S191F^* or *popdc1^KO^* on cardiac electrophysiology and Ca^2+^ handling in zebrafish larvae and in ex vivo adult hearts.

### 4.1. Summary of Results

Overall, we found that *popdc1^S191F^* and *popdc1^KO^* larvae had lower survival and higher development of a severe morphological phenotype than WT. In *popdc1^S191F^* or *popdc1^KO^* larvae that survived to 7 dpf and displayed a moderate morphological phenotype (involving spinal curvature and/or pericardial effusion), there were varied and highly variable, but consistent differences in cardiac electrophysiology and intracellular Ca^2+^ handling compared to WT, including lower HR, AV delay, and atrial and ventricular AP and CaT upstroke speed. In *popdc1^S191F^* larvae, these differences also included shorter atrial and ventricular AP duration but longer CaT duration. SNS stress with ISO increased HR and AV delay, slowed AP and CaT upstroke speed, and shortened AP and CaT duration in *popdc1^S191F^* or *popdc1^KO^* larvae displaying a morphological phenotype, but did not result in 2:1 AV block, as previously reported [12]. Some of the differences between WT and *popdc1^S191F^* or *popdc1^KO^* larvae remained during ISO exposure (i.e., lower HR, AV delay, and AP and CaT upstroke speed), but some were eliminated (i.e., shorter AP and longer CaT duration in the atrium and ventricle of *popdc1^S191F^* larvae, such that AP duration in the atrium of both *popdc1^S191F^* and *popdc1^KO^* became longer than WT).

V_m_ and Ca^2+^ dynamics before and during ISO exposure were also compared in *popdc1^S191F^* and WT adult isolated hearts. No differences in intracellular Ca^2+^ dynamics were found before ISO exposure, but in the atrium and ventricle AP upstroke speed was slower in *popdc1^S191F^* than WT, and in the ventricle, AP duration was longer. These differences between WT and *popdc1^S191F^* remained during ISO exposure, but ISO had differential effects on AP duration in the atrium and ventricle, as AP duration was increased in the atrium of WT hearts, while it was decreased in the ventricle of both WT and *popdc1^S191F^*. In addition, atrial CaT duration was decreased by ISO in *popdc1^S191F^* but not WT hearts, while ventricular CaT duration was decreased in both.

The most striking effect of *popdc1^S191F^* in adult hearts was on AVN function. 60.5% of all *popdc1^S191F^* hearts developed 2:1 AV block (42.1% during 2 Hz pacing and 38.9% during ISO exposure), compared to 20.0% of WT hearts (13.3% during 2 Hz pacing and 11.1% during ISO exposure, which is in line with the normal incidence of electrical instability in ex vivo adult zebrafish hearts reported by other labs [46]). The higher incidence of ISO-induced AV block in *popdc1^S191F^* hearts was accompanied by a difference in the effect of ISO on AV delay compared to WT; while AV delay was increased in both *popdc1^S191F^* and WT hearts, the resulting AV delay was greater in *popdc1^S191F^*.

### 4.2. Implications of Current Findings

In the present study, we have confirmed that *popdc1^S191F^* zebrafish have an arrhythmogenic phenotype, developing a 2:1 AV block similar to humans carrying the homologous *POPDC1^S201F^* mutation [12]. We did not, however, confirm previous results that this occurs in *popdc1^S191F^* larvae [12]. The difference between our results and the previous report may relate to the larvae studied. As we have shown here, the morphological phenotype in *popdc1^S191F^* larvae is broad, with some developing severe spinal curvature and/or pericardial effusion. In the present study, severely affected larvae were euthanised (to minimise possible confounding effects of overall embryo health status and to conform to ethical guidelines) and we examined 7 dpf larvae with normal morphology or a moderate morphological phenotype. In the previous study, larvae were examined at 5–9 dpf with a more severe morphological phenotype, which would be expected to have more severe electrical dysfunction. This is also reflected in differences in the reported impact on HR. In the previous study, 7 dpf *popdc1^S191F^* larvae with a severe morphological phenotype showed a ~50% lower HR than WT. It is likely that the larvae with a severe phenotype that were euthanised in our study would have had a similarly lower HR. Even so, the age-dependence of electrical dysfunction with POPDC mutation from larvae to adults found in our study is in line with what has been shown in mice [8,12] and in *POPDC1^S201F^* patients [12].

Further considering the phenotypic incidence in our study and its comparison to the previous report [12], we found that over the first 7 dpf in *popdc1^S191F^* larvae, there was considerably greater die-off (akin to embryonic lethality in mice) or development of a severe morphological phenotype requiring euthanasia than in WT, while in the previous study, at 5 dpf, “no significant lethality in comparison with WT embryos was observed”. Additionally, the incidence of morphological phenotypes in *popdc1^S191F^* vs. WT was greater in our study (13.1% and 0.8% incidence of moderate and severe phenotypes in *popdc1^S191F^* vs. 3.2% and 0.6% in WT, representing a 4.1× higher incidence of the moderate phenotype and a 3.7× higher incidence overall) than in the previous one (~25% and ~8% vs. ~10% and ~3%, representing a ~2.5× higher incidence for both). The greater lethality and incidence of a morphological phenotype in our study may be the result of several factors. We made measurements at 7 dpf from ~20 clutches, including ~2000 larvae per group, while the previous study made measurements at 5 dpf from only 3 clutches and ~200 larvae. As there is variability in survival and the incidence of morphological phenotypes between clutches, the nearly one order-of-magnitude lower number of clutches and larvae used in the previous study may have skewed the results. Also, as the incidence of morphological phenotypes varies with developmental stage, the difference in timing of the measurements may have had an impact. At the same time, phenotypic differences between the two studies may also have arisen from different environmental conditions or husbandry practices at the institutions where the studies were performed, as well as possible differences in the genetic load or background of the zebrafish. Environmental, husbandry, and genetic factors can result in a spectrum of phenotypes with different timing or levels of severity in animals carrying the same genetic variant.

Interestingly, in our study, we saw differences in the survival and cardiac effects between *popdc1^S191F^* and *popdc1^KO^* larvae. There was higher survival and less severe cardiac effects in *popdc1^S191F^*, as a lower HR compared to WT was only observed once a morphological phenotype was present, while in *popdc1^KO^* HR was already lower in animals in which a phenotype had not yet developed. HR was in fact higher than WT in *popdc1^S191F^* larvae without a morphological phenotype, which may represent an initial pathological effect of the mutation or a compensatory physiological response, which may have also occurred in the *popdc1^KO^* larvae, only at an earlier time point. These differences may not be surprising, though, as the timing and severity of the sub-cellular effects of the two mutations may differ. For instance, varied effects of POPDC1 mutations on proteostasis have been observed in patients carrying different genetic variants [14].

In the adult portion of our study, the incidence of *popdc1^S191F^* hearts in which 2:1 AV block was induced by ISO was likely reduced by the exclusion of ex vivo hearts that developed AV block with 2 Hz pacing. If those hearts could have been included, many would likely have been affected by SNS stress. Even still, twice as many *popdc1^S191F^* hearts developed 2:1 AV block during ISO than WT. This higher incidence of AV block in *popdc1^S191F^* was associated with a greater increase in AV delay, which suggests that dysfunction in SNS control of AVN function in *popdc1^S191F^* may play a role in the occurrence of AV block. The source of this dysfunction requires further study—for which the zebrafish may be a useful model—and may be directly related to impacts of POPDC1 on β-AR function, cAMP nanodomain signaling and sub-cellular compartmentalisation, or disturbances in the function, trafficking, or localisation of interacting proteins [4]. The predominance of an effect of SNS stress on the AVN in our study, rather than on the SAN (as observed in mice carrying mutations of *Popdc1*), may, in part, relate to the greater relative density of sympathetic to parasympathetic nerves within the AVN (compared to the SAN) in zebrafish [47].

Ventricle-specific effects of *popdc1^S191F^* on V_m_ dynamics may also have played a part in the occurrence of AV block, as ventricular AP upstroke speed was slower and AP duration longer in *popdc1^S191F^* hearts compared to WT, which may have led to failed ventricular excitation due to reduced excitability and/or increased refractoriness. Disruption of the POPDC1-phosphodiesterase 4 complex—which prevents premature cAMP binding of POPDC1 under basal conditions—has been shown to prolong AP duration in rabbit isolated ventricular myocytes (possibly due to decreased interaction between POPDC1 and TREK1 and probably other ion channels [7]). Such a disruption of protein–protein interaction may also occur in zebrafish carrying *popdc1* variants. Interestingly, two genome-wide association studies have identified several single nucleotide polymorphisms that affect the expression level of *POPDC1* and possibly are associated with human long QT syndrome [13,48]. Comparing our results to previous murine investigations, in *popdc1^KO^* mice it was found that—like in our study—CaT upstroke speed was reduced [49], while forced expression of Popdc1*^S201F^* in murine HL-1 cardiac muscle cells—in contrast to our results—AP upstroke speed was increased [12], further highlighting species differences that should be considered in future studies.

In the zebrafish larvae studied, we observed impacts of *popdc1* mutations on V_m_ and Ca^2+^ dynamics, as well as effects of ISO; however, these were not severe enough to induce arrhythmias. Surprisingly, we found a shorter AP duration in *popdc1^S191F^* larvae, but this is in fact similar to what has been reported for zebrafish *popdc1* morphants [13]. The absence of SNS-induced AV block in *popdc1^S191F^* larvae, compared to its presence in adult hearts, may relate to the divergent effect of ISO on AV delay; in larvae, ISO caused a decrease in AV delay, while in adult hearts, AV delay was increased. The reasons for this disparity require further study, but may relate to: (i) ongoing development of the AVN [41,50] or cardiomyocyte function [51] in larval zebrafish; (ii) ongoing development of cardiac innervation or β-AR expression [52,53,54]; or (iii) differences in the effects of ISO between the in vivo setting (in which the heart is still under central nervous system control) and the ex vivo setting (in which the heart has been disconnected from the central nervous system). In the larval hearts in our study, AP and CaT upstroke speeds were slower than in the adult, indicating that cardiac electrophysiology and intracellular Ca^2+^ handling may not be fully developed, and thus may not be affected in the same way by SNS stress. Developmental differences in the response to SNS stress may also relate to differences in the heart’s innervation or expression of autonomic receptors. In the adult zebrafish heart, changes in nerve density, the ratio of sympathetic to parasympathetic nerves, and the V_m_ and Ca^2+^ handling response to sympatho-vagal nerve stimulation have been shown to occur up to 12 mpf [34]. In zebrafish larvae, the expression of autonomic receptors and response to autonomic stimulation is dependent on the developmental stage [52,53,54]. Pharmacological autonomic agonists affect HR as early as ~4 dpf, while direct cardiac innervation is not believed to be fully established until ~11 dpf [54]. Therefore, it is likely that the ISO responses we observed in 7 dpf larvae are the result of direct stimulation of β-AR on cardiomyocytes, while the responses in ex vivo adult hearts may result from the direct stimulation of myocardial receptors as well as neuronal receptor stimulation activating intracardiac neuronal feedback loops [55].

### 4.3. Study Limitations

While we have shown the utility of the zebrafish for understanding *popdc1*-related arrhythmogenic mechanisms, as with any experimental model one must consider limitations that may mitigate the translation of findings to humans. While almost every cardiac gene in the zebrafish has a human ortholog with analogous function, including the POPDC genes [30], and cardiac electrophysiology is more similar to humans than rodents in many ways important for arrhythmogenesis [21,22,23,24,25], the zebrafish heart: (i) is smaller than the human or rodent heart; (ii) has only two (rather than four) chambers; (iii) operates at relatively low intra-cardiac pressure [43]; (iv) has a greater dependency of contraction on trans-sarcolemmal Ca^2+^ flux than SR Ca^2+^ release [25]; (v) has myocytes lacking a well organised T-tubular system [56]; (vi) has a reduced (or significantly different) conduction system [57]; and (vii) has a degree of genetic redundancy due to genome duplications that may compensate for effects of some genetic mutations (although the latter does not apply to the POPDC gene family, which consists of three POPDC genes in zebrafish and in mammals) [30]. Even still, in the current study, zebrafish revealed the impacts of a *popdc1* mutation on the cardiac AP, CaT, and AVN function, which may play a role in the observed SNS-induced 2:1 AV block. SNS stress in our study, however, was pharmacological, targeting β-AR on cardiomyocytes. This response may differ from central nervous system-driven SNS activation, so future experiments should investigate the effects of direct cardiac nerve stimulation. A further consideration important for future studies is the impact of *popdc1* dysfunction on the general health status of the zebrafish larvae studied. While *popdc1* larvae with a normal morphology or a moderate morphological phenotype (spinal curvature and/or pericardial effusion) were selected in the current study, and larvae displaying a severe morphological phenotype were excluded, a general decline in health may in part account for the observed effects on the heart’s electrophysiology and intracellular Ca^2+^ handling, rather than cardiac-specific effects.

### 4.4. Future Directions

In the present study, we have investigated the impact of *popdc1* loss-of-function mutations on V_m_ and Ca^2+^ dynamics in 7 dpf zebrafish larvae and in hearts isolated from 18–22 mpf adults. We have shown that impaired AVN function or ventricular V_m_ dynamics may underly the development of AV block with *popdc1* mutation; however, the underlying molecular mechanisms were not explored. Future work involving genetic manipulation of genes important for AV conduction, the ventricular AP, or those known to interact with POPDC1 may help reveal novel therapeutic targets.

We observed a greater lethality and incidence of a morphological phenotype in *popdc1^S191F^* larvae compared to the previous study [12], as well as differences in the survival and cardiac effects of *popdc1^S191F^* and *popdc1^KO^*. Even in people or animals carrying the same genetic variant, it is often observed that individuals display a spectrum of phenotypes with different levels of severity or age of onset (even in genetically identical twins). These differences may relate to environmental factors (e.g., body weight, diet, exercise), which, in combination with mutant alleles, potentiate a pathological phenotype. Even with identical environmental conditions, though, some mutations lead to a varied pathological phenotype, driven by differences in genetic background (other genetic variants or modifier genes). It is possible that there exist modifiers interacting with *popdc1* in zebrafish that result in differential phenotype expression. Given the high genetic tractability of the zebrafish, exploring potential influences on the effects of *popdc1* dysfunction represents an exciting opportunity for future research.

It would also be pertinent to determine the timing of the development of V_m_ and Ca^2+^ dysfunction, by studying zebrafish at intermittent time points, while at the same time establishing the effects of *popdc1* dysfunction in the hearts of older, aged zebrafish. The importance of atrial versus ventricular *popdc1* expression in zebrafish should also be explored, as in the mammalian heart, there is a greater expression of POPDC1 in the atria than ventricles [8], but whether this applies to the zebrafish is unknown. We did not consider potential sex differences in the response to *popdc1* dysfunction, and while we are not aware of any reported sex differences in zebrafish cardiac function, sex differences in the function and molecular mechanisms of cardiac pacemaking have been described in other species [58], so should be considered in future studies. A final possibly exciting target for future investigation involves defining the importance of POPDC1 in neurons of the intracardiac nervous system, which may be an upstream mediator of detrimental electrophysiological effects in cardiomyocytes, given the role of SNS-induced arrhythmogenesis with *popdc*1 mutation, and that POPDC1 proteins are present in the brain, spinal cord, and dorsal root ganglia [5,59]. The presence of POPDC1 in other neuronal populations, and the abundance of vital POPDC1-interacting proteins in intracardiac neurons, suggests a potentially important role for POPDC1 proteins in these cells.

## 5. Conclusions

Our zebrafish experimental model has revealed the effects of two *popdc1* loss-of-function mutations on AVN function and cardiac electrophysiology and intracellular Ca^2+^ handling that may underlie arrhythmogenesis observed in patients. This suggests that the zebrafish may be a useful tool in future studies exploring underlying molecular mechanisms to identify targets for novel anti-arrhythmic therapies.

## Figures and Tables

**Table 1:** **Cardiac effects of *popdc1* dysfunction in larval zebrafish.** Measured values from WT, *popdc1^S191F^* (S191F), and *popdc1^KO^* (KO) zebrafish larvae with no morphological phenotype or with a moderate phenotype. Values reported as mean ± SEM.

Larvae Pre ISO					
		No Phenotype		Phenotype	
	WT	S191F	KO	S191F	KO
**HR (bpm)**	244 ± 2	252 ± 2	228 ± 2	227 ± 4	231 ± 3
**AV Delay (ms)**	70.0 ± 1.5	61.8 ± 1.3	52.2 ± 1.2	54.3 ± 2.3	54.4 ± 1.2
**AP Upstroke Speed (dF_n_/dt_max_, s^−1^)**					
** Atrium**	21.4 ± 0.7	22.3 ± 0.7	24.7 ± 0.6	18.1 ± 0.5	18.4 ± 0.5
** Ventricle**	19.3 ± 0.7	20.2 ± 0.6	20.4 ± 0.7	16.6 ± 0.5	16.6 ± 0.4
**APD_80_ (ms)**					
** Atrium**	151 ± 4	162 ± 4	171 ± 6	137 ± 5	153 ± 5
** Ventricle**	192 ± 4	186 ± 6	187 ± 6	165 ± 4	209 ± 5
**CaTD Upstroke Speed (dF_n_/dt_max_, s^−1^)**					
** Atrium**	23.3 ± 0.3	24.3 ± 0.3	24.6 ± 0.3	19.2 ± 0.3	20.7 ± 0.4
** Ventricle**	17.4 ± 0.2	17.8 ± 0.2	17.6 ± 0.2	15.3 ± 0.3	15.6 ± 0.3
**CaTD_80_ (ms)**					
** Atrium**	169 ± 2	169 ± 2	173 ± 3	183 ± 3	170 ± 3
** Ventricle**	193 ± 1	187 ± 2	207 ± 2	206 ± 4	203 ± 3

**Table 2:** **Effects of ISO on cardiac function in larval zebrafish.** Measured values from WT, *popdc1^S191F^* (S191F), and *popdc1^KO^* (KO) zebrafish larvae with a moderate morphological phenotype. Values reported as mean ± SEM.

Larvae during ISO			
	WT	S191F	KO
**HR (bpm)**	262 ± 2	251 ± 3	243 ± 3
**AV Delay (ms)**	73.5 ± 1.0	64.8 ± 1.3	60.8 ± 1.4
**AP Upstroke Speed (dF_n_/dt_max_, s^−1^)**			
** Atrium**	17.6 ± 0.5	15.6 ± 0.5	15.4 ± 0.5
** Ventricle**	15.8 ± 0.5	13.7 ± 0.4	13.3 ± 0.4
**APD_80_ (ms)**			
** Atrium**	113 ± 4	128 ± 4	127 ± 4
** Ventricle**	172 ± 3	165 ± 4	175 ± 4
**CaTD Upstroke Speed (dF_n_/dt_max_, s^−1^)**			
** Atrium**	23.3 ± 0.4	17.8 ± 0.4	17.4 ± 0.5
** Ventricle**	17.3 ± 0.2	13.1 ± 0.3	14.7 ± 0.3
**CaTD_80_ (ms)**			
** Atrium**	153 ± 2	157 ± 2	167 ± 3
** Ventricle**	181 ± 2	180 ± 2	188 ± 3

**Table 3:** **Effects of ISO on cardiac function in adult isolated zebrafish hearts.** Measured values from WT and *popdc1^S191F^* (S191F) zebrafish hearts. Values reported as mean ± SEM.

Adult				
	WT		S191F	
**AV Delay (ms)**	50.1 ± 3.0	62.7 ± 3.5	51.6 ± 4.7	87.1 ± 6.6
**AP Upstroke Speed (dF_n_/dt_max_, s^−1^)**				
** Atrium**	148.1 ± 4.4	144.9 ± 6.0	134.7 ± 4.4	136.1 ± 3.0
** Ventricle**	132.7 ± 5.7	132.1 ± 6.7	106.0 ± 5.0	101.8 ± 5.9
**APD_80_ (ms)**				
** Atrium**	67 ± 3	74 ± 4	71 ± 7	73 ± 7
** Ventricle**	170 ± 5	164 ± 5	196 ± 8	187 ± 6
**CaTD Upstroke Speed (dF_n_/dt_max_, s^−1^)**				
** Atrium**	83.4 ± 4.7	81.8 ± 5.8	94.3 ± 2.8	94.2 ± 2.4
** Ventricle**	90.9 ± 5.3	91.1 ± 5.5	89.5 ± 2.5	79.8 ± 5.6
**CaTD_80_ (ms)**				
** Atrium**	133 ± 8	153 ± 47	127 ± 4	121 ± 4
** Ventricle**	261 ± 16	241 ± 15	253 ± 12	228 ± 12

## Data Availability

The raw data supporting the conclusions of this article will be made available by the authors upon reasonable request.
